# RAPD2: Rapid Automated Processing of Macromolecular Crystallographic Data 2

**DOI:** 10.1063/4.0001069

**Published:** 2025-10-27

**Authors:** Jonathan P Schuermann, Kay Perry, David Neau, Frank V. Murphy

**Affiliations:** Cornell University, Ithica, NY, USA

## Abstract

RAPD2 is a modular package of programs written for the automated processing of macromolecular crystallographic data at the NE-CAT beamlines. It monitors for collected data, processes snapshots to create strategies for data collection, processes data runs for structure solution, and can then solve the structure using molecular replacement or single-wavelength anomalous diffraction with results stored in a MongoDB. Most of the backend code is written in Python3 with an AngularJS based frontend. This allows users to login with a web browser to view results, modify settings and rerun jobs, or launch additional pipelines (see Kay Perry). The RAPD2 code is designed to be modular on multiple levels. With a variety of possible experimental and computing environments in mind, RAPD2 is separated into several interdependent modules. At the highest level, X-Ray source monitoring (Monitors), core data handling and archiving (Control), and job launching (Launch) can be started through the Control or separated into distinct programs that communicate by passing Python objects over standard TCP sockets or Redis Streams. This allows flexibility in setup; for example, RAPD was developed on a single computer, so the Control and the Launch programs initially ran on a single machine; when a computational cluster was later acquired, the Launch module was moved with minimal changes to either program. On a deeper level, the code is divided into functionally distinct modules. For example, each pipeline is self-contained and called by a launch adapter when needed, allowing nimble development (*i.e.* bug fixes) that do not require any program restarting to take effect. Additional benefits of the built-in modularity are that all site-specific settings and functions are isolated as much as possible to one module (Site), so adapting RAPD to a new experimental environment is relatively simple.

At NE-CAT, Redis Streams are used for communication between the beamline and the RAPD2 Monitors; however this can be modified to suit different environments at other facilities. The Monitors save the beamline information in MongoDB and pass it to Control, where the strategy or data processing commands are generated. These commands are sent to a Launch manager that decides where to launch the job based on Site settings. This provides flexibility in launching jobs on specific machines using a shell launch adapter or on a computer cluster with launch adapters for various cluster workload managers including SLURM, SGE, and PBS. For strategy commands, the index pipeline is launched, where six Labelit autoindexing jobs are started simultaneously with different peak pick settings, each optimized for different types of diffraction data. Once the best solution is determined, Raddose is run to calculate radiation damage parameters, which are input to BEST for the regular and anomalous data collection strategies. This pipeline takes approximately 30s to complete, and results are displayed in the UI. The index pipeline is modular, so other auto-index, radiation damage, or strategy programs can be launched through different plugins. For data runs, the integration pipeline is launched running XDS multiple times to optimize the results. This pipeline takes a few minutes to finish, and results are displayed in the UI. A new mintegrate pipeline will additionally launch data processing in autoPROC, Fast DP, and XIA2, with results from all 4 data processing programs displayed in the UI for comparison.

